# SOX17 expression and its down‐regulation by promoter methylation in cervical adenocarcinoma in situ and adenocarcinoma

**DOI:** 10.1111/his.13980

**Published:** 2019-12-01

**Authors:** Anton N H Hopman, Jobran M Moshi, Klaas J Hoogduin, Monique Ummelen, Mieke E R Henfling, Manon van Engeland, Kim A D Wouters, Hans Stoop, Leendert H J Looijenga, Frans C S Ramaekers

**Affiliations:** ^1^ Department of Molecular Cell Biology GROW School for Oncology & Developmental Biology Maastricht University Medical Centre Maastricht the Netherlands; ^2^ Department of Medical Laboratory Technology Faculty of Applied Medical Sciences Jazan University Jazan Kingdom of Saudi Arabia; ^3^ Laboratory for Experimental Patho‐Oncology Department of Pathology Erasmus University Medical Centre Rotterdam the Netherlands; ^4^ Department of Pathology GROW School for Oncology & Developmental Biology Maastricht University Medical Centre Maastricht the Netherlands; ^5^ Princess Maxima Centre for Paediatric Oncology Utrecht the Netherlands; ^6^ Present address: Pathan B.V. Sint Franciscus Gasthuis Rotterdam the Netherlands

**Keywords:** cervical (pre)neoplasia, keratins, reserve cells, SOX2, SOX17, squamocolumnar junction, transformation zone, uterine cervix

## Abstract

**Aims:**

SOX17 expression has not been studied in glandular lesions of the uterine cervix like adenocarcinoma in situ (AIS) and invasive adenocarcinomas (AdC), whereas *SOX17* promoter CpG island methylation has been reported. Therefore, the aim of this study was to relate the topographical distribution of SOX17 expression and *SOX17* methylation status to each other, and to SOX2 expression, human papillomavirus (HPV) type, and physical status of the virus.

**Methods and results:**

Immunohistochemistry was used in 45 cases to assess expression of SOX17 and SOX2. *SOX17* promoter methylation was determined in 25 cases by means of bisulphite conversion and methylation‐specific polymerase chain reaction. SOX17 and SOX2 showed a mutually exclusive expression pattern in normal epithelium, with a sharp delineation in the squamocolumnar junction. SOX17 was found in endocervical columnar and reserve cells, whereas SOX2 was exclusively found in squamous epithelium. In both glandular lesions and cases with coexisting glandular and squamous intraepithelial components, a complex combination of SOX17 and SOX2 expression patterns was seen and mutually exclusive expression was lost. Frequently, gain of expression of SOX2 was found and expression of SOX17 was lost. Methylation of the CpG island in the *SOX17* promoter was shown to be strongly associated with loss of expression of SOX17 (*P* = 0.0016).

**Conclusions:**

In this study, we show for the first time a direct correlation between the topographical distribution of SOX17 expression and the methylation status of its gene promoter. This explains the heterogeneity of SOX17 expression in the glandular lesions of the cervix. No correlation was found between HPV type and physical status of the virus on the one hand and methylation status on the other.

## Introduction

Cervical cancer is the fourth most frequent cancer in women, with an estimated 570 000 new cases in 2018. Approximately 90% of deaths from cervical cancer occurred in low‐income and middle‐income countries.^1^ Human papillomavirus (HPV) has been identified as a definite human carcinogen for six types of cancer, i.e. cancers of the cervix, penis, vulva, vagina, anus, and oropharynx (including the base of the tongue and tonsils).^2^, ^3^ Cervical cancer originates in the transformation zone of the uterine cervix through HPV infection. In this dynamic area, premalignant lesions originate and endocervical columnar cells are replaced by squamous epithelium. The squamocolumnar junction (SqCJ) is the landmark between these two epithelial tissue types.^4^, ^5^, ^6^, ^7^, ^8^, ^9^ There is a general consensus that reserve cells can be regarded as a progenitor cell population capable of generating squamous‐type epithelium,^9^ but can also differentiate into columnar cells, thereby replenishing the endocervical glandular epithelium.^5^, ^10^, ^11^, ^12^, ^13^ However, direct evidence for this type of cell being a progenitor for normal columnar epithelium is still lacking. Recently, Mockler *et al*., using keratin 17 (K17) immunohistochemistry, showed a phenotypic correlation between these subcolumnar reserve cells and glandular neoplasia.

They reported that K17 was not found in the epithelial cells of benign glandular lesions, but rather in groups of cuboidal cells residing beneath the epithelial layer of these lesions. The majority of endocervical adenocarcinoma (AdC) and AdC in situ (AIS) is K17‐positive, suggesting a role for these cells in the development of such endocervical (pre)malignancies.^14^, ^15^

Both squamous and glandular carcinomas develop in the cervical transformation zone which is an important argument for the hypothesis that the reserve cells play a central role in the pathogenesis of both squamous and glandular cervical lesions.^16^, ^17^, ^18^, ^19^, ^20^, ^21^

The majority of cervical cancers are represented by squamous cell carcinomas (SCCs) (~80%) and AdCs (~20%), developing through their respective premalignant lesions, i.e. squamous intraepithelial lesion (SIL) and AIS. Coexistence of SIL and AIS can be found in the same specimen.^13^, ^19^

Viral integration has been reported as an early event in AIS formation, as opposed to SIL, in which precancerous lesions showed exclusively episomal viral genomes, and a minority of the carcinomas had integrated viral genomes.^16^, ^22^, ^23^

HPV16, HPV18 and HPV45 were found substantially more often in the integrated state than HPV31 and HPV33. HPV promotes cellular proliferation and abrogates cell cycle checkpoints involving p53 and retinoblastoma protein (pRb). These proteins are deregulated by the viral E6 and E7 proteins, and viral integration results in constant expression of these proteins. This is a result of the inactivation of the viral E2 protein by its deletion, which leads to enhancement of E6 and E7 expression, influencing tumour progression.^2^

In the current study, we immunohistochemically examined the expression pattern of the transcription regulator protein SRY‐box containing gene 17 (SOX17) in normal cervical glandular epithelium and in the lesions derived therefrom. Only HPV‐associated glandular lesions were studied.

SOX17 is known to play a pivotal role in human development,^24^, ^25^ but so far its distribution in the uterine cervix has not been studied. The promoter CpG island region of *SOX17* has been reported to be methylated in the majority of squamous and glandular (pre)malignant lesions of the uterine cervix.^26^ As a single marker, promoter CpG island methylation of SOX17 has been shown to be associated with progression of high‐grade SIL (HSIL) into carcinoma.^27^ Another transcription regulator protein from the SRY‐box gene family, SOX2, has been shown to play an important role during the progression of SIL lesions. The expression level increased with severity of the SIL lesion.^28^, ^29^ This marker has not yet been studied in AIS and AdC.

Recently, growing evidence has indicated that SOX17 plays an important role in human carcinogenesis. Down‐regulation of SOX17 expression has been detected in colorectal cancer, hepatocellular carcinoma, gastric cancer, lung and oesophageal carcinoma, among others.^30^, ^31^, ^32^ Hypermethylation of the *SOX17* promoter correlates with a poor prognosis for several cancers, and provides important prognostic information in breast cancer patients.^32^ Recent studies have shown that SOX17 is also epigenetically silenced in circulating tumour cells isolated from the peripheral blood of patients with breast or gastric cancer, and can be used as a molecular diagnostic marker in early‐stage gastric cancer.^32^

In the current study, we therefore also examined the role of *SOX17* promoter CpG island methylation in the development of glandular lesions of the uterine cervix. SOX2 expression, HPV typing of the lesions, chromogenic in‐situ hybridisation (CISH) and several molecular markers were used for correlation with SOX17 expression and *SOX17* promoter methylation.

## Materials and methods

### Tissues

The following types of tissue sample were selected from the archives of the Departments of Pathology of the Foundation of Collaborating Hospitals in Eastern Groningen, Pathan Rotterdam and the Reinier de Graaf Hospital Delft, The Netherlands:

1. Formalin‐fixed paraffin‐embedded (FFPE) tissues from 12 normal cervices removed for non‐cervix‐related conditions during hysterectomy of premenopausal women. In 11 of the 12 samples, reserve cells could be found. There was no previous history of cervical abnormalities.
2. FFPE tissues from representative samples of cervical (pre)neoplastic lesions were selected, including 23 AIS cases. In 10 of these AIS cases, a coexisting SIL was recognised; two were low‐grade SIL (LSIL) and eight were HSIL. Furthermore, 22 endocervical AdCs were examined. For comparison, nine LSIL cases, 23 HSIL cases and 20 SCC cases were also included. Sections were re‐evaluated by two pathologists, who selected representative tissue blocks in each case. Regions with microglandular hyperplasia were occasionally found in these samples. In all of these cases, SOX17 expression was assessed with immunohistochemistry, and 11 solitary AISs, five lesions with coexisting AIS and SIL components, nine AdCs, eight LSILs and 10 HSILs were also tested for methylation of *SOX17*. These were all analysed for HPV, and selected cases were additionally immunostained for several differentiation markers.

Research on tumour samples was performed according to the Code for Proper Secondary Use of Human Tissue in The Netherlands (://www.federa.org/, update 2011), and was approved by the Medical Ethics Committees of the Erasmus University Medical Centre, Rotterdam and of the Foundation of Collaborating Hospitals of Eastern Groningen, The Netherlands.

### Immunohistochemistry

Immunohistochemical staining on 4‐µm‐thick FFPE tissue sections was performed with primary antibodies against SOX17, SOX2, K17, Ki67, and p16. Detailed information on antibodies and assay conditions is given in Table S1 in Data S1. The sections were scanned with a Ventana iScan HT slide scanner (Ventana Medical Systems, Tucson, AZ, USA), and semiquantitatively scored for expression of SOX17, SOX2, K17, Ki67, and p16. Images were viewed and selected with image viewer Software (Ventana Medical Systems).

### HPV

HPV genotyping was performed with the multiplex ligation‐dependent probe amplification assay or the Single tube Multiplex Amplification in Real Time kit (PathoFinder, Maastricht, The Netherlands).^33^, ^34^ HPV typing was performed on DNA isolated from 4‐µm‐thick FFPE tissue sections or DNA isolated from cytological samples matched with the tissue obtained after colposcopy and processed for histological examination.

### HPV In‐Situ Hybridisation (ISH)

HPV was detected by means of CISH, with DNA probes for HPV16, HPV18, HPV31, and HPV45. For a detailed description of HPV ISH, see Data S1. The CISH signals were classified according to their distribution patterns, which were typical for the presence of episomal viral copies, viral replication, or viral integration into the human genome.^35^

### *SOX17* Promoter CpG Island Methylation

DNA was isolated from cells from FFPE sections by means of manual dissection. The areas for isolation were selected on the basis of p16 and SOX17 immunostaining, or ISH HPV positivity (Data S1). DNA was bisulphite‐treated (EZ DNA Methylation‐Direct Kit; Zymo Research, Irvine, CA, USA), and, after conversion, the potentially methylated *SOX17* DNA was amplified. Subsequently, polymerase chain reaction with specific primers for the methylated CpG island and unmethylated CpG island was performed (Table S2 in Data S1).^36^, ^37^ To compare the methylation status with the immunohistochemical expression of SOX17, Fisher’s exact test was used to test the statistical significance.

## Results

### Expression Patterns of SOX17 In The Normal Adult Cervix

**Figure 1:**
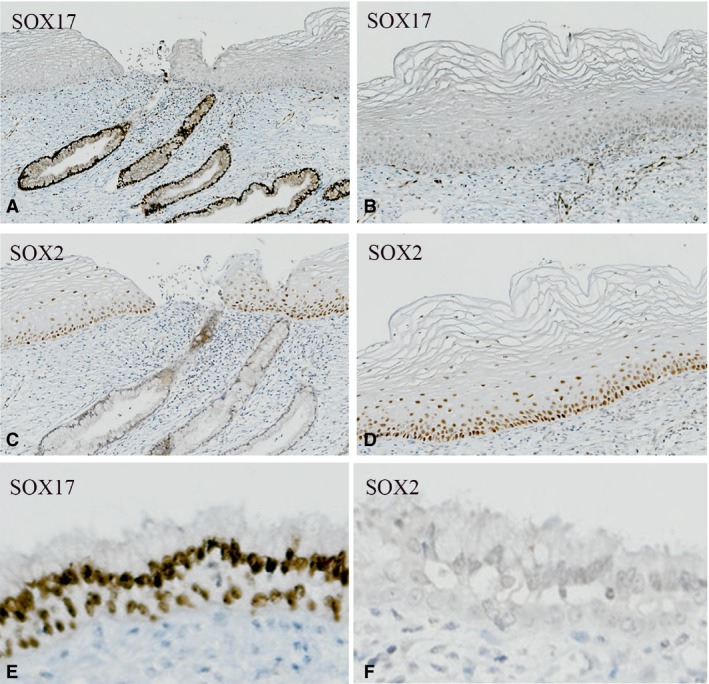
Mutually exclusive SOX17 and SOX2 immunostaining of normal ectocervical and endocervical epithelium. SOX17 staining (**A**,**B**,**E**) and SOX2 staining (**C**,**D**,**F**) in corresponding areas show SOX17 positivity and SOX2 negativity in the normal glandular invaginations (**A**,**C**), and the opposite staining pattern in mature squamous epithelium (**B**,**D**). The reserve cells underlying the glandular epithelium are only SOX17‐positive (**E**,**F**).

In samples of the normal adult cervix, the abrupt mucosal transition between the squamous epithelium and the endocervical epithelium the SqCJ was characterised by the exclusive expression of SOX17 in the glandular (columnar) epithelium (Figure 1A) and its absence in the normal ectocervical squamous epithelium (Figure 1B). SOX17 was also extensively expressed in the reserve cells underlying the columnar epithelium (Figure 1E). SOX17 was occasionally found in the cases with mature squamous metaplasia (result not shown). SOX2 expression is, in general, an indicator of squamous differentiation, and was therefore used to immunophenotype these cell types in the normal cervix. A mutually exclusive staining pattern of SOX2 and SOX17 was seen, with mainly the basal and suprabasal layers of the squamous epithelium being positive for SOX2, and no reactivity in the glandular component (compare Figure 1C,D). Reserve cell SOX2 expression could not be found (Figure 1F).

### Expression Patterns of SOX17 In Glandular (PRE) Malignant Lesions

**Figure 2:**
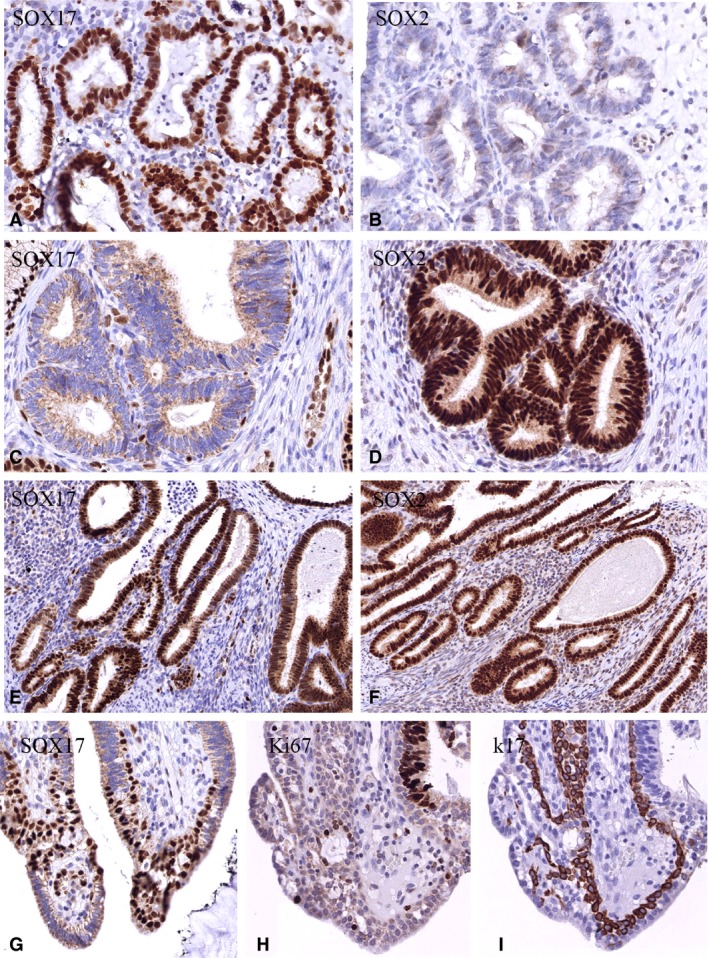
Examples of the three different combinations of SOX17 and SOX2 expression patterns found in solitary adenocarcinoma in situ (AIS). **A**,**B**, Only strong nuclear SOX17 positivity and SOX2 negativity (a few nuclei stained weakly for SOX2). **C**,**D**, Only strong nuclear SOX2 positivity and nuclear negativity for SOX17. **E**,**F**, Strong simultaneous expression of SOX2 and SOX17. **G**,**H**,**I**, High reserve cell density (SOX17 and keratin 17 positivity, and Ki67 negativity) in close proximity to AIS.

The expression patterns of SOX17 and SOX2 were semiquantitatively assessed in total 35 glandular lesions (AIS and AdC), and 10 lesions with coexisting AIS and SIL components (Table 1). For comparative reasons, in total 52 squamous lesions, like LSIL, HSIL and SCC, were also analysed (results not shown). Many of the endocervical glandular (pre)malignant lesions, which were expected to express exclusively SOX17 (see, for example, Figure 2A,B), on the basis of the above‐described results for the normal cervical epithelium, showed coexpression of SOX17 and SOX2 (Figure 2E,F), or even exclusive expression of SOX2 (Figure 2C,D). In 21 of 35 solitary AISs and AdCs, SOX17 expression was evident, and in 18 of these lesions SOX2 expression was found. In four cases, both SOX17 and SOX2 were expressed to a comparable extent, and five cases were negative for both. K17‐positive reserve cells were found in small areas of AIS lesions, and showed strong nuclear SOX17 positivity and no Ki67 staining (Figure 2G–I).

**Table 1:** Overview of SOX17 and SOX2 expression patterns in glandular lesions of the uterine cervix

Glandular lesions	SOX17+ only	SOX2+ only	SOX17+/SOX2+	SOX17−/SOX2−
Solitary AIS (*n* = 13)	6	5	2	0
Coexisting AIS and SIL (*n* = 10)				
AIS	5	1	3	1
SIL	2	7	1	0
Adenocarcinomas (*n* = 22)	6	4	7	5

AIS, Adenocarcinoma in situ; SIL, Squamous intraepithelial lesion.

**Figure 3:**
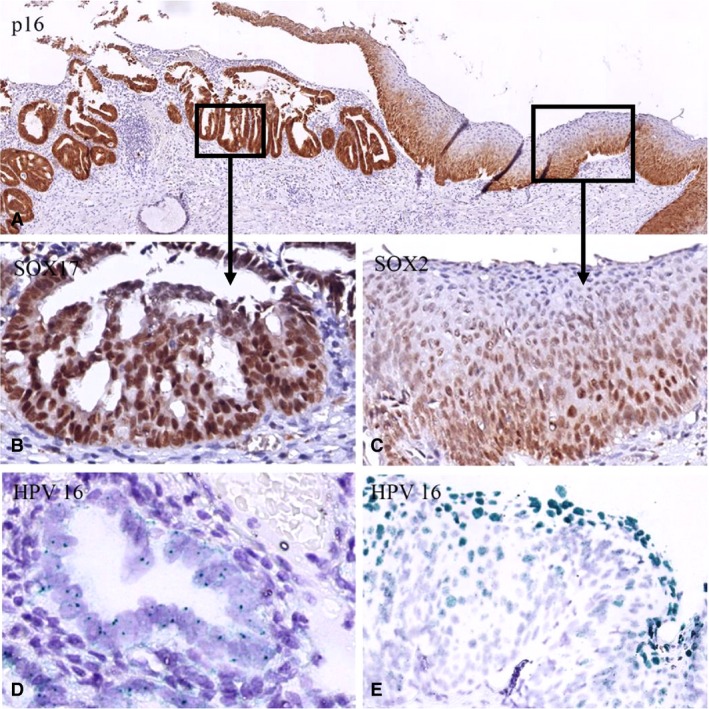
Example of a premalignant lesion with coexisting glandular and squamous components. **A**, Low magnification of a lesion with coexisting adenocarcinoma in situ (AIS) and squamous intraepithelial lesion (SIL) components immunostained for p16 (case 13). **B**, Glandular component expressing SOX17. **C**, Squamous component expressing SOX2. **D**,**E**, The human papillomavirus 16 (HPV16) chromogenic in‐situ hybridisation patterns show HPV16 integration in the AIS component (**D**) and replication of the virus in the low‐grade SIL component (**E**).

In lesions with coexisting glandular and squamous premalignant components (AIS/SIL; Figure 3), the glandular component in eight of 10 synchronous lesions expressed predominantly SOX17 (Figure 3B), whereas the squamous component exclusively expressed SOX2 in seven of the 10 cases (Figure 3C). One case was negative for both, and two SIL lesions showed predominant SOX17 expression. In all of these cases, HPV could be found (Table 2, coexisting cases), with two cases showing viral replication in the SIL component, and the AIS component showing the virus to be integrated (Figure 3D,E).

**Table 2:**
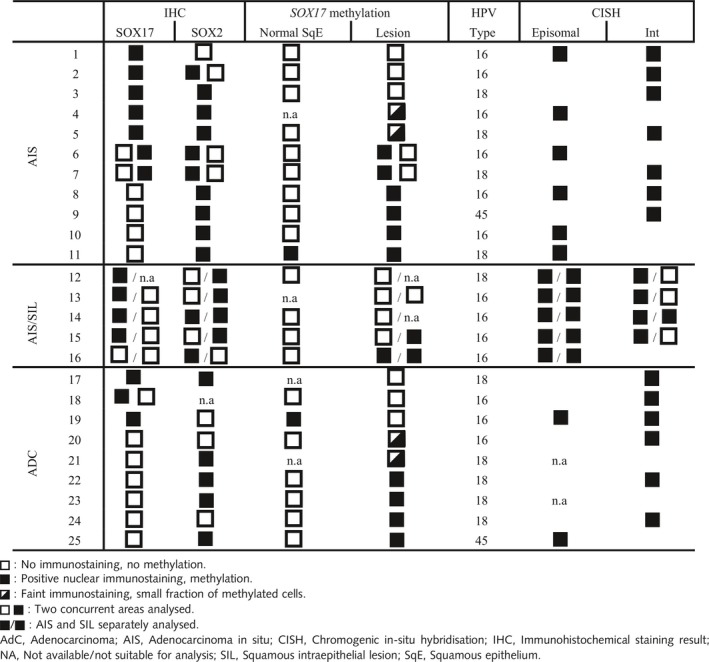
Overview of SOX17 and SOX2 expression in relation to *SOX17* methylation status, human papillomavirus (HPV) type in glandular lesions, and physical status of the virus

For reference purposes, SIL lesions and cervical SCCs were also immunostained for SOX17 and SOX2. Exclusive SOX2 positivity or predominant expression of SOX2 was seen in 27 of the 32 SIL lesions (LSIL and HSIL), and only two cases showed predominant or exclusive expression of SOX17. Coexpression of SOX17 with SOX2 was found in six of 32 cases. All 20 SCCs showed exclusive SOX2 expression in 30–100% of the cells (results not shown).

### Promoter CpG Island Methylation Is Associated With Down‐Regulation Of SOX17 Expression In Glandular Lesions

**Figure 4:**
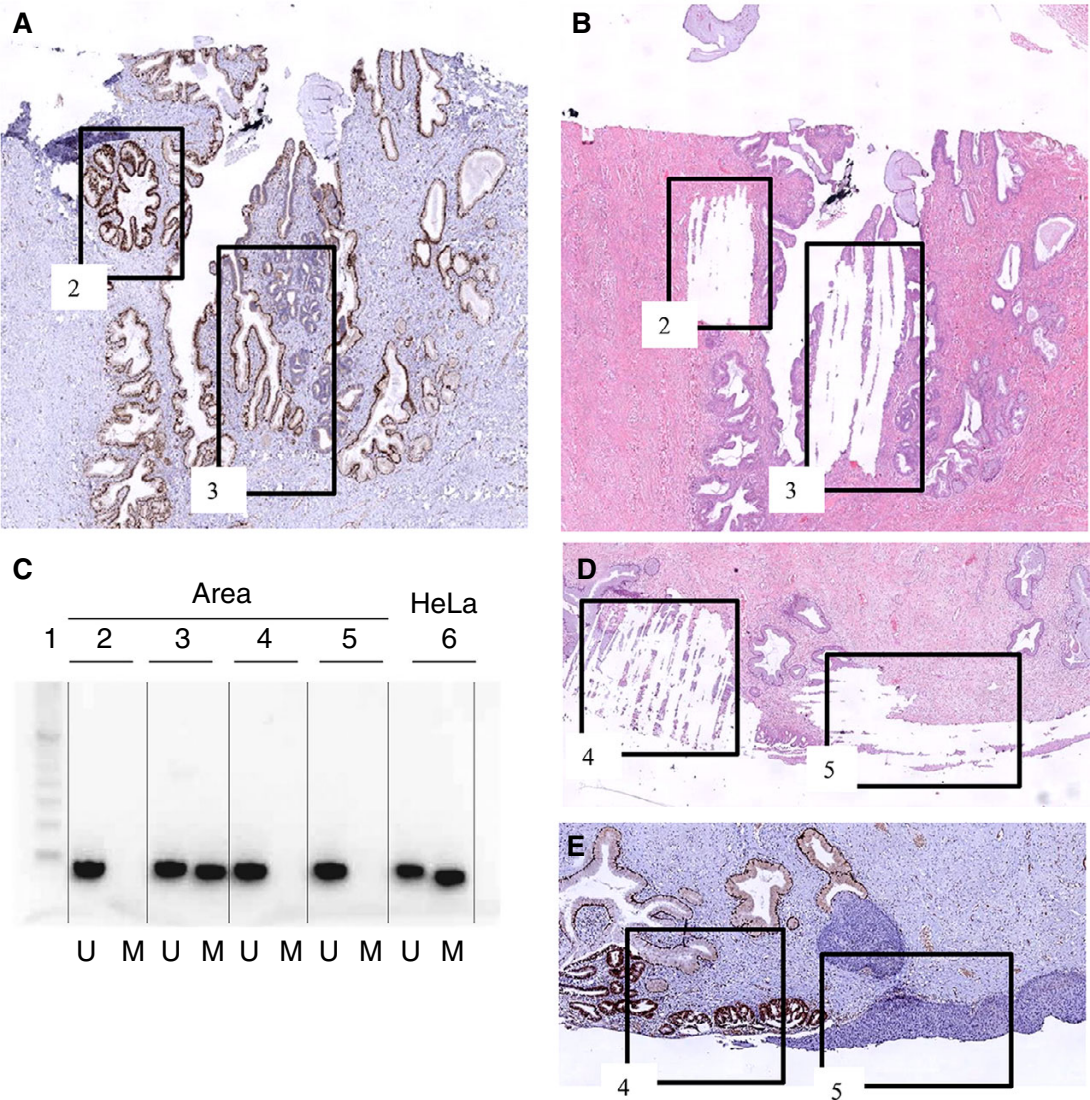
Examples of methylation analyses of *SOX17* in a solitary adenocarcinoma in situ (AIS) and a lesion with coexisting AIS and squamous intraepithelial lesion (SIL) components. **A**,**B**, Solitary AIS (case 6). **D**,**E**, Lesion with coexisting AIS and SIL components (case 13). (**A**) and (**E**) show SOX17 immunohistochemical staining of these lesions. Tissue was isolated by means of manual scraping (**B**,**D**). DNA was isolated from two dysplastic areas within AIS case 6 (area 2, SOX17‐positive; area 3, SOX17‐negative) and from two areas within AIS/SIL case 13 (area 4, AIS SOX17‐positive; area 5, high‐grade SIL SOX17‐negative). **C**, Methylation status of *SOX17* in the different areas with an internal positive DNA control (HeLa) in lane 6. U, unmethylated CpG island; M, methylated CpG island.

To explain the unexpected loss of SOX17 expression in the AISs and AdCs, the promoter CpG island methylation status of *SOX17* in these lesions was determined. In both AIS and AdC methylation of this promoter region was strongly associated with down‐regulation of SOX17 expression at the protein level (Table 2). In 10 cases, methylation could be found when SOX17 was not expressed, whereas, in seven cases, SOX17‐positive areas were non‐methylated. In three of these cases, an abrupt change in SOX17 immunostaining was seen in adjacent regions within the same lesion. In two of these cases (cases 6 and 7 in Table 2), the methylation status could be analysed (see Figure 4A–C for illustration of case 6), and showed the association between SOX17 expression and *SOX17* methylation status. Fisher’s exact test was used to test the statistical significance; a *P*‐value of 0.0016 was determined for the association between non‐methylated CpG islands in the promoter region of *SOX17* and expression of SOX17 versus promoter methylation and loss of expression of SOX17 in glandular preneoplasia in a total of 32 analysed areas. In the lesions with coexisting AIS and SIL components (cases 12–16 in Table 2), the association between methylation status and expression of SOX17 was confirmed in all samples (see also Figure 4C–E).

In normal squamous epithelium, used as a control, the examined CpG island in the promoter region of *SOX17* was methylated in only one of 15 samples examined, whereas, in six of 18 SIL cases, methylation of *SOX17* was found, mostly in HSIL (results not shown).

### CISH

Table 2 provides an overview of HPV type and physical status of the virus. Thirteen cases were HPV16‐positive, 10 cases were HPV18‐positive (eight could be analysed with CISH), and two cases were HPV45‐positive. HPV16 was found to be integrated in nine of 13 (AIS and AdC) lesions, and HPV18 was found to be integrated in seven of eight cases. HPV45 was integrated in one case. Among the lesions with coexisting AIS and SIL components, all cases showed episomal copies, whereas, in four of five AIS cases, the virus was shown to be integrated. We noted that four of seven and two of nine cases showed combined methylation of *SOX17* and viral integration for, respectively, HPV18 and HPV16.

## Discussion

The two major types of premalignant cervical lesion are SIL, which represents 80% of cases, and AIS with combined AIS/SIL lesions, which represents ~20% of cases. The transformation zone between the glandular epithelium and the squamous epithelium of the cervix has been described as a high‐risk region for the development of these lesions.^8^, ^9^, ^11^, ^12^, ^38^ We have now shown that the mutually exclusive expression patterns of the transcription factors SOX17 and SOX2 mark the SqCJ in the normal transformation zone of the adult cervix. Their specific immunostaining patterns distinguish between the SOX2‐positive squamous epithelium on the one hand, and the SOX17‐positive endocervical glandular epithelium on the other. Reserve cells were shown to express SOX17 but not SOX2. The fact that columnar and reserve cells share SOX17 expression suggests that the latter can act as the progenitor cells of endocervical columnar epithelium. It could also be an equally strong argument for the concept that columnar cells are progenitors of the reserve cells, as suggested by Crum *et al.* 39; such a transition has also been shown to occur during human embryogenesis.^40^

On the basis of the SOX expression patterns seen in normal endocervical columnar cells and in reserve cells, we expected that AIS and AdC would exclusively express SOX17. Indeed, approximately half of the glandular lesions expressed SOX17, whilst the other half were SOX17‐negative, matching perfectly with *SOX17* promoter methylation.

Earlier studies have found the promoter CpG island region of *SOX17* to be methylated in the majority of squamous and glandular lesions of the uterine cervix,^26^ and this was also shown to be associated with progression of HSIL to carcinoma.^27^ In the current study, we directly correlated the topographical SOX17 distribution pattern, as determined with immunohistochemistry, with epigenetic changes, i.e. methylation of the *SOX17* promoter region. This combination of techniques allows us to draw some specific conclusions. For example, in individual cases, abrupt loss of SOX17 as seen in adjacent AIS regions suggests that the lesion progresses from an unmethylated *SOX17* status to a methylated *SOX17* status. However, we observed no significant correlation between progression of AIS to AdC and down‐regulation of SOX17 expression.

At the molecular level, the epigenetic silencing of *SOX17* has been correlated with activation of the Wnt signalling pathway. Sinner *et al*.^41^ and Jia *et al*.^31^ have shown that SOX17 promotes the degradation of β‐catenin–T‐cell factor (TCF) via a glycogen synthase kinase‐3β‐independent mechanism, and Van der Meide *et al*.^26^ have suggested that there is direct inhibition of β‐catenin–TCF–lymphoid enhancer factor function by SOX17. Recently, Li *et al*.^42^ also demonstrated that SOX17 restrains proliferation and tumour formation in squamous cervical cancer by down‐regulating the activity of the Wnt–β‐catenin signalling pathway via trans‐suppression of β‐catenin. Therefore, down‐regulation of SOX17 expression by promoter methylation can result in activation of the Wnt signalling pathway, stimulating the proliferation and genetic instability of premalignant lesions. This also suggests that, in SOX17‐positive regions of AIS, alternative pathways induce proliferation.

The evident pathway that promotes cellular proliferation and abrogates cell cycle checkpoints involves p53 and pRb via HPV. These proteins are deregulated by the viral E6 and E7 proteins. Viral integration into the human genome results in constant expression of these proteins, as the E2 protein is inactive because of deletion. As a result, E6 expression and E7 expression are promoted, which subsequently promotes the development of the neoplastic lesion.^2^

Statistical analysis did not reveal a significant association between the *SOX17* methylation status of the lesions and viral integration. Viral integration has been reported for HPV18 in adenomatous lesions, as confirmed in our study, and HPV16 was integrated in more than half of the lesions.^22^, ^43^

We can only conclude that *SOX17* methylation and viral integration, although frequently detected simultaneously in glandular lesions, are independent processes. Also, no direct interaction between viral proteins and *SOX17* promoter regions or transcription regulator proteins has been reported so far.

The expression of SOX2 in a major proportion of the solitary AIS and AdC cases was unexpected. This may be a reflection of the bidirectional differentiation properties of the reserve cell. Furthermore, the expression of SOX2 also supports our earlier findings^19^ that solitary AIS can express markers of squamoid differentiation such as keratin 5 and p63 or could be the result of the gain of chromosome 3q harbouring the *SOX2* gene.^44^, ^45^ AIS has been reported to be associated with SIL in 25–75% of cases.^13^, ^16^, ^19^, ^46^ The concurrence of both types of lesion in the same biopsy supports the hypothesis that both lesions may arise from a single progenitor cell or area at risk. Reserve cells, which are capable of undergoing both columnar and squamous differentiation, may be candidate progenitor cells for the formation of these combined lesions.^10^, ^19^, ^39^ This view is supported by the observation that, in incidental cases of lesions with coexisting AIS and SIL, K17‐positive reserve cells were found to be p16‐positive and HPV‐positive.^10^

In conclusion, this study shows, for the first time, a direct correlation between the topographical distribution of SOX17 expression and the methylation status of its gene promoter region. This combination of techniques has clearly shown the heterogeneity of SOX17 expression in the glandular lesions of the cervix, an observation that could not be made on the sole basis of the methylation studies performed so far.

## Conflict of interest

The authors have no conflicts of interest.

## Author Contribution

**Hopman AHN**: writing; evaluation of all IHC reactions, methylation data, FISH / CISH results and imaging. **Moshi Jobran M**: writing; sorting data for AIS; literature search; interpretation FISH/CISH data. **Hoogduin KJ**: selections of (pre)neoplasia, analysis of IHC staining patterns for SOX2, SOX17; conceptual discussions. **Ummelen M**: technical performance of IHC staining p16, FISH and CISH and imaging. **Henfling MER**: technical performance of IHC staining of part of sections for SOX2 and SOX17; imaging. **Manon van Engeland**: Dept. Pathology (Pathobiology of Cancer, in particular the role of Epigenetics), responsible for KW. Conceptual discussions. **Kim A.D Wouters**: design primers and methylation assay of SOX17. **Stoop H**: IHC staining of part of sections for SOX2 and SOX17. **Looijenga LHJ**: As head of Dept. Experimental Patho‐Oncology final responsibility for SH, responsible for SOX staining. Conceptual discussions. **Ramaekers FCS**: As head of the Dept. Molecular Cell Biology final responsibility for team AH, MU, MH. Conceptual discussions and writing. All persons read the manuscript and wrote part for which they were responsible (for technical performance: see above).

## Supporting information


**Data S1** **.** Supplementary materials and methods.
**Table S1** **.** Antibody characteristics and optimised immunohistochemical methods.
**Table S2** **.** Primer design.

## References

[1] WHO . Cervical cancer. *Bull World Health Organ* 2018 Available at: https://www.who.int/cancer/prevention/diagnosis-screening/cervical-cancer/en/.

[2] Woodman CB , Collins SI , Young LS . The natural history of cervical HPV infection: unresolved issues. Nat. Rev. Cancer 2007; 7; 11–22. 17186016 10.1038/nrc2050

[3] Forman D , de Martel C , Lacey CJ *et al* Global burden of human papillomavirus and related diseases. Vaccine 2012; 30; F12–F23. 23199955 10.1016/j.vaccine.2012.07.055

[4] Wright TC , Ferenczy A . Benign diseases of the cervix. 6th ed. New York: Springer‐Verlag, 2002; 253–324.

[5] Mukonoweshuro P , Oriowolo A , Smith M . Audit of the histological definition of cervical transformation zone. J. Clin. Pathol. 2005; 58; 671–690. 15917428 PMC1770692

[6] Schiffman M , Castle PE , Jeronimo J , Rodriguez AC , Wacholder S . Human papillomavirus and cervical cancer. Lancet 2007; 370; 890–907. 17826171 10.1016/S0140-6736(07)61416-0

[7] Herfs M , Hubert P , Delvenne P . Epithelial metaplasia: adult stem cell reprogramming and (pre)neoplastic transformation mediated by inflammation? Trends Microbiol. 2009; 15; 245–253. 10.1016/j.molmed.2009.04.002 19457719

[8] Herfs M , Hubert P , Moutschen M , Delvenne P . Mucosal junctions: open doors to HPV and HIV infections? Trends Microbiol. 2011; 19; 114–120. 21216598 10.1016/j.tim.2010.12.006

[9] Reich O , Regauer S , McCluggage WG , Bergeron C , Redman C . Defining the cervical transformation zone and squamocolumnar junction: can we reach a common colposcopic and histologic definition? Int. J. Gynecol. Pathol. 2017; 36; 517–522. 28639968 10.1097/PGP.0000000000000381

[10] Smedts F , Ramaekers F , Troyanovsky S *et al* Basal‐cell keratins in cervical reserve cells and a comparison to their expression in cervical intraepithelial neoplasia. Am. J. Pathol. 1992; 140; 601–612. 1372156 PMC1886162

[11] Burghardt E , Ostör AG . Site and origin of squamous cervical cancer: a histomorphologic study. Obstet. Gynecol. 1983; 62; 117–127. 6856213

[12] Hopman AHN , Ramaekers FCS . Development of the uterine cervix and its implications for the pathogenesis of cervical cancer. 3rd ed. Edinburgh: Springer Internatinal Publishing AG, 2017.

[13] Bekkers RL , Bulten J , Wiersma‐van Tilburg A *et al* Coexisting high‐grade glandular and squamous cervical lesions and human papillomavirus infections. Br. J. Cancer 2003; 89; 886–890. 12942122 10.1038/sj.bjc.6601204 PMC2394485

[14] Mockler D , Escobar‐Hoyos LF , Akalin A , Romeiser J , Shroyer AL , Shroyer KR . Keratin 17 is a prognostic biomarker in endocervical glandular neoplasia. Am. J. Clin. Pathol. 2017; 148; 264–273. 28821199 10.1093/ajcp/aqx077

[15] Escobar‐Hoyos LF , Yang J , Zhu J *et al* Keratin 17 in premalignant and malignant squamous lesions of the cervix: proteomic discovery and immunohistochemical validation as a diagnostic and prognostic biomarker. Mod. Pathol. 2014; 27; 621–630. 24051697 10.1038/modpathol.2013.166 PMC4026928

[16] Witkiewicz A , Lee KR , Brodsky G , Cviko A , Brodsky J , Crum CP . Superficial (early) endocervical adenocarcinoma in situ: a study of 12 cases and comparison to conventional AIS. Am. J. Surg. Pathol. 2005; 29; 1609–1614. 16327433 10.1097/01.pas.0000173239.24955.a2

[17] Brown LJ , Wells M . Cervical glandular atypia associated with squamous intraepithelial neoplasia: a premalignant lesion? J. Clin. Pathol. 1986; 39; 22–28. 3950029 10.1136/jcp.39.1.22 PMC499608

[18] Regauer S , Reich O . CK17 and p16 expression patterns distinguish (atypical) immature squamous metaplasia from high‐grade cervical intraepithelial neoplasia (CIN III). Histopathology 2007; 50; 629–635. 17394499 10.1111/j.1365-2559.2007.02652.x PMC1890920

[19] Smedts F , Ramaekers FC , Hopman AH . The two faces of cervical adenocarcinoma in situ. Int. J. Gynecol. Pathol. 2010; 29; 378–385. 20567153 10.1097/PGP.0b013e3181cd3175

[20] Kerdraon O , Cornelius A , Farine MO , Boulanger L , Wacrenier A . Adenoid basal hyperplasia of the uterine cervix: a lesion of reserve cell type, distinct from adenoid basal carcinoma. Hum. Pathol. 2012; 43; 2255–2265. 22809729 10.1016/j.humpath.2012.03.023

[21] Schiffman M , Doorbar J , Wentzensen N *et al* Carcinogenic human papillomavirus infection. Nat. Rev. Dis. Primers 2016; 2; 16086. 27905473 10.1038/nrdp.2016.86

[22] Vinokurova S , Wentzensen N , Kraus I *et al* Type‐dependent integration frequency of human papillomavirus genomes in cervical lesions. Cancer Res. 2008; 68; 307–313. 18172324 10.1158/0008-5472.CAN-07-2754

[23] Hopman AH , Theelen W , Hommelberg PP *et al* Genomic integration of oncogenic HPV and gain of the human telomerase gene TERC at 3q26 are strongly associated events in the progression of uterine cervical dysplasia to invasive cancer. J. Pathol. 2006; 210; 412–419. 17054308 10.1002/path.2070

[24] de Jong J , Stoop H , Gillis AJ *et al* Differential expression of SOX17 and SOX2 in germ cells and stem cells has biological and clinical implications. J. Pathol. 2008; 215; 21–30. 18348160 10.1002/path.2332

[25] Julian LM , McDonald AC , Stanford WL . Direct reprogramming with SOX factors: masters of cell fate. Curr. Opin. Genet. Dev. 2017; 46; 24–36. 28662445 10.1016/j.gde.2017.06.005

[26] van der Meide WF , Snellenberg S , Meijer CJ *et al* Promoter methylation analysis of WNT/beta‐catenin signaling pathway regulators to detect adenocarcinoma or its precursor lesion of the cervix. Gynecol. Oncol. 2011; 123; 116–122. 21726894 10.1016/j.ygyno.2011.06.015

[27] Hansel A , Steinbach D , Greinke C *et al* A promising DNA methylation signature for the triage of high‐risk human papillomavirus DNA‐positive women. PLoS One 2014; 9; e91905. 24647315 10.1371/journal.pone.0091905 PMC3960142

[28] Ji J , Zheng PS Expression of SOX2 in human cervical carcinogenesis. Hum. Pathol. 2010; 41; 1438–1447. 20709360 10.1016/j.humpath.2009.11.021

[29] Liu XF , Yang WT , Xu R , Liu JT , Zheng PS . Cervical cancer cells with positive Sox2 expression exhibit the properties of cancer stem cells. PLoS One 2014; 9; e87092. 24489842 10.1371/journal.pone.0087092 PMC3904967

[30] Zhang W , Glockner SC , Guo M *et al* Epigenetic inactivation of the canonical Wnt antagonist SRY‐box containing gene 17 in colorectal cancer. Cancer Res. 2008; 68: 2764–2772. 18413743 10.1158/0008-5472.CAN-07-6349 PMC2823123

[31] Jia Y , Yang Y , Liu S * et al* SOX17 antagonizes WNT/β‐catenin signaling pathway in hepatocellular carcinoma. Epigenetics 2010; 5; 743–749. 20716954 10.4161/epi.5.8.13104

[32] Fu D , Ren C , Tan H * et al* Sox17 promoter methylation in plasma DNA is associated with poor survival and can be used as a prognostic factor in breast cancer. Medicine 2015; 94; e637. 25789956 10.1097/MD.0000000000000637 PMC4602484

[33] Theelen W , Litjens RJ , Vinokurova S * et al* Human papillomavirus multiplex ligation‐dependent probe amplification assay for the assessment of viral load, integration, and gain of telomerase‐related genes in cervical malignancies. Hum. Pathol. 2013; 44; 2410–2418. 23968641 10.1016/j.humpath.2013.04.026

[34] Litjens RJ , Theelen W , van de Pas Y *et al* Use of the HPV MLPA assay in cervical cytology for the prediction of high grade lesions. J. Med. Virol. 2013; 85; 1386–1393. 23765775 10.1002/jmv.23629

[35] Hopman AH , Kamps MA , Smedts F , Speel EJ , Herrington CS , Ramaekers FC . HPV in situ hybridization: impact of different protocols on the detection of integrated HPV. Int. J. Cancer 2005; 115; 419–428. 15688369 10.1002/ijc.20862

[36] Derks S , Lentjes MH , Hellebrekers DM , de Bruine AP , Herman JG , van Engeland M . Methylation‐specific PCR unraveled. Cell Oncol. 2004; 26; 291–299. 15623939 10.1155/2004/370301 PMC4611123

[37] Gao L , van den Hurk K , Moerkerk PTM *et al* Promoter CpG island hypermethylation in dysplastic nevus and melanoma: CLDN11 as an epigenetic biomarker for malignancy. J Invest Dermatol 2014; 134; 2957–2966. 24999589 10.1038/jid.2014.270

[38] Mirkovic J , Howitt BE , Roncarati P *et al* Carcinogenic HPV infection in the cervical squamo‐columnar junction. J. Pathol. 2015; 236; 265–271. 25782708 10.1002/path.4533 PMC4457596

[39] Witkiewicz AK , Hecht JL , Cviko A , McKeon FD , Ince TA , Crum CP *et al* Microglandular hyperplasia: a model for the de novo emergence and evolution of endocervical reserve cells. Hum. Pathol. 2005; 36; 154–161. 15754292 10.1016/j.humpath.2004.10.017

[40] Martens JE , Smedts F , van Muyden RC *et al* Reserve cells in human uterine cervical epithelium are derived from mullerian epithelium at midgestational age. Int. J. Gynecol. Pathol. 2007; 26; 463–468. 17885499 10.1097/pgp.0b013e31803c7c18

[41] Sinner D , Rankin S , Lee M *et al* Sox17 and β‐catenin cooperate to regulate the transcription of endodermal genes. Development 2004; 131; 3069–3080. 15163629 10.1242/dev.01176

[42] Li L , Yang WT , Zheng PS , Liu XF . SOX17 restrains proliferation and tumor formation by down‐regulating activity of the Wnt/β‐catenin signaling pathway via trans‐suppressing β‐catenin in cervical cancer. Cell Death Dis. 2018; 9; 741–750. 29970906 10.1038/s41419-018-0782-8 PMC6030085

[43] Holl K , Nowakowski AM , Powell N *et al* Human papillomavirus prevalence and type‐distribution in cervical glandular neoplasias: results from a European multinational epidemiological study. Int. J. Cancer 2015; 137; 2858–2868. 26096203 10.1002/ijc.29651 PMC5034816

[44] Andersson S , Wallin KL , Hellström AC *et al* Frequent gain of the human telomerase geneTERC at 3q26 in cervical adenocarcinomas. Br. J. Cancer 2006; 95; 331–338. 16847471 10.1038/sj.bjc.6603253 PMC2360637

[45] Thomas LK , Bermejo JL , Vinokurova S *et al* Chromosomal gains and losses in human papillomavirus‐associated neoplasia of the lower genital tract–a systematic review and meta‐analysis. Eur. J. Cancer 2014; 50; 85–98. 24054023 10.1016/j.ejca.2013.08.022

[46] Horn LC , Klostermann K . Precancerous lesions of the uterine cervix: morphology and molecular pathology. Pathologe 2011; 32(Suppl 2); 242–254. 21909794 10.1007/s00292-011-1517-0

