# Supplementary material for: SOX17 expression and its down‐regulation by promoter methylation in cervical adenocarcinoma in situ and adenocarcinoma
Source: Histopathology. 2019 Dec 1;76(3):383–93. doi: 10.1111/his.13980 (PMC7027543; doi:10.1111/his.13980)
Supplement: Supplementary file 1 — Data S1 . Supplementary materials and methods. Table S1 . Antibody characteristics and optimised immunohistochemical methods. Table S2 . Primer design. [file HIS-76-383-s001.docx]

**Supporting Information.**

Supplementary Materials and Methods:

**Immunohistochemistry**

After standard deparaffinization in xylene and blocking of endogenous peroxidase activity by 0.3% peroxide in methanol, the 4 µm thick tissue sections were boiled in 10 mM Tris-EDTA buffer pH 9.0 for 20 min in a microwave oven for antigen retrieval. The antigens were detected with the primary antibodies, secondary antibodies and enhancement procedures as listed in Supplementary Table 1, stained with diaminobenzidine.HCl (DAB) and finally counterstained with hematoxylin.

**S1 Table: Antibody characteristics and optimized immunohistochemical methods.**

| **Antigen** | **Primary Antibody** | **Dilution** | **Secondary antibody** | **Enhancement/Detection** |
| --- | --- | --- | --- | --- |
| SOX2 | pAb Goat IgG  AF2018  R&D Systems, Abbingdon,UK | 1:750 in PBST/1% BSA,  1 hr RT | Biotinylated Horse anti Goat IgG, Vector Laboratories, Burlingame, CA, USA,  BA-9500  1:200 in PBST/1% BSA; 30 min RT | ABC, Vectastain Elite ABC Kit, Vector Laboratories, Burlingham, CA, USA,  30 min RT  DAB reaction |
| SOX17 | pAb Goat IgG  GT15094  Neuromics, Edina, MN, USA | 1:2500 in PBST/1% BSA,  1 hr RT | Biotinylated Horse anti Goat IgG, Vector Laboratories,  BA-9500  1:200 in PBST/1% BSA; 30 min RT | ABC  30 min RT  DAB reaction |
| p16 | mAb Mouse IgG  Clone E6H4  CINtec, MTM Laboratories AG, Heidelberg, Germany | 1:50 in PBST/1% BSA,  1 hr RT | Biotinylated Horse anti Mouse IgG, Vector Laboratories,  BA-2001  1:200 in PBST/1% BSA; 30 min RT | ABC  30 min RT  DAB reaction |
| k17 | mAb Mouse IgG  Clone E3  MUB0325P  Nordic-MUbio, Susteren, The Netherlands | 1:200 in PBST/1% BSA,  1 hr RT | Poly-HRP Goat anti Mouse/Rabbit IgG, Immunologic, Duiven, The Netherlands,  Undiluted  30 min 37°C | None  DAB reaction |
| Ki67 | mAb Mouse IgG1  Clone MIB-1  Dako, Glostrup, Denmark | 1:25 in  PBST/1% BSA,  1 hr RT | Biotinylated Horse anti Mouse IgG, Vector Laboratories,  BA-2001  1:200 in PBST/1% BSA; 30 min RT | ABC  30 min RT  DAB reaction |

Abbreviations: mAb, monoclonal antibody; pAb, polyclonal antibody; HRP, horseradish peroxidase; RT, room temperature; PBST, phosphate buffered saline + 0.1% Tween-20 (Janssen Chimica, Beerse, Belgium); BSA, bovine serum albumin; DAB, diaminobenzidine; ABC, Avidin-biotin complex with biotinylated HRP.

***SOX17* methylation analysis**

DNA isolation and bisulfite conversion

The 4 µm thick FFPE tissue sections were deparaffinized in xylol, washed with 100% ethanol and subsequently air dried. Based on hematoxylin and eosin (H&E) staining or specific immunohistochemical staining patterns, cells from the lesions were manually dissected using the edge of an 18X18 cm coverslip (Menzel-Gläser, Braunschweig, Germany). From every sample normal squamous epithelial cells were dissected as controls (see Figure 4). The tissue samples were transfered to 26 µl digestion buffer containing Proteinase K (following the instruction of the EZ DNA Methylation-Direct Kit; Zymo Research, Irvine, CA, USA) and incubated for 4 hours at 50°C. Bisulfite conversion was performed by adding 20 µl of digested sample to 130 µl of the C to T (CT) conversion reagent. After incubation for 8 minutes at 98°C and 3.5 hours at 64°C in a thermal cycler, the samples were transferred to a Zymo-Spin IC column, containing binding buffer. After centrifugation and washing of the samples, desulphonation buffer was added to the columns. Following centrifugation and two washing steps, the DNA was eluted in 10 µl of elution buffer.

Methylation specific PCR

Methylation specific primer (MSP) design and MSP analysis on bisulfite-treated DNA was performed as described previously ^1^. Since only small regions from the FFPE sections were dissected and the DNA yield was expected to be low, the DNA was first amplified with flanking PCR primers, serving as a template for MSP analysis with the specified primers. All PCRs were performed with controls for both methylated DNA (normal human placenta DNA treated in vitro with Sssl methyltransferase (New England Biolabs, Ipswich, MA, USA)) and unmethylated DNA (Epitect unmethylated DNA, Qiagen, Venlo, The Netherlands). The SOX 17 methylation-specific PCR (MSP) primers are located at chromosome 8 (BLAST and BiSearch genomic region 54458315-54458421) and shown in Supplementary Table 2.

DNA from HeLa cells, which shows bands for methylated and unmethylated DNA was incorporated as a control sample, as well as water controls. Flanking PCRs were performed in a volume of 25 µl containing 200 nM of each primer, 312.5 nM dNTPs (GE Healthcare Europe GmbH, Eindhoven, The Netherlands), 0.5 U Immolase DNA polymerase (Bioline, London, UK) and 4 µl of bisulfite treated DNA. Amplification was performed in a Thermocycler UNO II (Biometra GmbH, Göttingen, Germany), starting with an initial denaturation step of 3 min at 95°C, followed by 35 cycles of 95°C, 56°C and 72°C for 30 sec each, with a final extension step of 4 min at 72°C. A second PCR with primers specific for methylated or unmethylated *SOX17* (see Supplementary Table 2) was performed in a volume of 25 µl containing 400 nM of each primer, 62.5 nM dNTPs, 0.5 U of Immolase DNA polymerase and 4 µl of diluted amplified DNA resulting from the flanking PCR. PCR conditions were as follows: 10 min at 95°C, followed by 35 cycles of 95°C, 56°C and 72°C for 30 sec each, with a final extension step of 4 min at 72°C. PCR reaction products were resolved on 2% agarose gels with GelStar nucleic acid gel stain (Cambrex Bio Science Rockland, Inc, Rockland, ME, USA) and visualized under UV light. Bands with approximately equal intensity for methylated and unmethylated DNA were scored positive. Faint methylated bands were analyzed again and if persistent considered negative for methylation ^2^.

|  | **Primer** | **Sense primer (5’→3’) Antisense primer (5’→3’)** | **Annealing temp. (°C)** | **PCR cycles**  **(No.)** |
| --- | --- | --- | --- | --- |
|  |  |  |  |  |
| *SOX17* | Flank | TGTGTAGGTTTGGATTTTGTTG AACCRAACCAAAAACRAATCC | 56°C | 35 |
|  | U | ATTTTGTTGTGTTAGTTGTTTGTGTTT ACAAATCCCATATCCAACAACCA | 60°C | 35 |
|  | M | TTGCGTTAGTCGTTTGCGTTC TCCCGTATCCGACGACCG | 60°C | 35 |

**S2 Table: Primers Design**.

**In Situ Hybridization**

Chromogenic (CISH) in situ hybridization: The probes for HPV 16, HPV 18, HPV 31 (PanPath, Uden, The Netherlands) and HPV 45 (Dr. E . de Villiers, Deutsches Krebsforschungs Zentrum, Heidelberg, Germany) were labeled with biotin by standard nick translation and used at a concentration of 1 ng/µl in a solution containing 50% formamide, 2 × SSC, 10% dextran sulphate and 50 × excess of carrier DNA (salmon sperm DNA; Sigma, Steinheim, Germany) ISH was performed on 4 µm thick FFPE tissue sections fixed onto Superfrost Plus Microscope Slides (Thermo Fisher Scientific). In brief, to improve adhesion to the slides during the ISH procedure, tissue sections were first heated for 15 min at 80°C. Following heating, the sections were deparaffinized in xylol, hydrated and microwaved for 10 min at 100°C in a 10 mM Na-Citrate pH 6.0 buffer, and incubated at room temperature for 20 min to cool down. Subsequently, the sections were washed in demineralized water, rinsed in 10 mM HCl and digested with 2.0 mg/ml pepsin (601 units/mg: porcine gastric mucosa, Sigma, Steinheim, Germany) in 10 mM HCl. Thereafter, the slides were washed once in 10 mM HCl, and once in PBS and post-fixed in 1% formaldehyde in PBS for 5 min at room temperature. After the post-fixation, the slides were washed with PBS, demineralized water and dehydrated in an ascending alcohol series. Subsequently, the HPV probe was applied under a coverslip, simultaneously denatured for 10 min at 80°C and hybridized overnight at 37°C. After hybridization, the slides were washed for 5 min at 42°C in a solution containing 2 × SSC, 0.05% Tween-20 (Janssen Chimica, Beerse, Belgium) and subsequently washed twice for 5 min at 61°C in 0.1 × SSC.

The hybridized probe was detected in a triple layer detection method with peroxidase-conjugated avidin (1: 100 dilution; Vector Laboratories, CA, USA), biotinylated goat anti-avidin (1:100 dilution: Vector Laboratories) and finally peroxidase-conjugated avidin. The enzymatic reaction was performed using Vina Green according to the instructions of the supplier (Vina Green Chromogen Kit, BIOCARE Medical, CA, USA). Finally, the slides were washed in milliQ, counterstained with hematoxylin, immediately dehydrated and embedded in Entellan new,(Merck, Darmstadt,Germany). Images were recorded with the Nikon Eclipse E800 (mounted with a color camera) and Nikon ACT-1 software. HPV physical status as concluded from the ISH patterns was classified as episomal or integrated, and with or without a typical replication pattern ^3^.

**References to Supplementary Materials and Methods**

1. Derks S, Lentjes MH, Hellebrekers DM, *et al.* Methylation-specific PCR unraveled. *Cell Oncol* 2004; **26**; 291-299.

2. Gao L, van den Hurk K, Moerkerk PTM, *et al.* Promoter CpG island hypermethylation in dysplastic nevus and melanoma: CLDN11 as an epigenetic biomarker for malignancy. *J* *Invest Dermatol* 2014; **134**; 2957-2966.

3. Hopman AH, Kamps MA, Smedts F, *et al.* HPV in situ hybridization: impact of different protocols on the detection of integrated HPV. *Int J Cancer* 2005;**115**;419-428.
